# MR Imaging of Stem Cell Transplants in Arthritic Joints

**DOI:** 10.4172/2157-7633.1000165

**Published:** 2014-02-07

**Authors:** Heike E Daldrup-Link, Hossein Nejadnik

**Affiliations:** Department of Radiology and Molecular Imaging Program at Stanford (MIPS), Stanford School of Medicine, 725 Welch Rd, Rm 1665; Stanford, USA

**Keywords:** SPIO, MR imaging techniques, MASI

## Abstract

About 43 million individuals in the US currently suffer from disabilities due to arthritis. Cartilage defects are the major source of pain in the affected joints. Current treatments, whilst alleviating some of the clinical symptoms, prove insufficient to cure the underlying irreversible cartilage loss. Stem cells represent a unique source for restoration of cartilage defects. Pre-clinical and clinical trials are currently pursued to investigate the potential of various types of stem cells and stem cell derived chondrocytes to repair arthritic joints. A major challenge with all stem cell-mediated tissue regeneration approaches is death of the transplanted cells with clearance by the immune system. Our current inability to diagnose successful or unsuccessful engraftment of transplanted cells non-invasively *in vivo* represents a major bottleneck for the development of successful stem cell therapies. A large variety of non-invasive Magnetic Resonance (MR) imaging techniques have been developed over the last decade, which enable sensitive *in vivo* detection of Matrix Associated Stem Cell Implants (MASI) and early diagnosis of related complications. While initially focused on successfully harvesting cellular MR imaging approaches with easily applicable SuperParamagnetic Iron Oxide Nanoparticles (SPIO), our team began to observe details that will facilitate clinical translation. We therefore started a broader effort to define a comprehensive set of novel, clinically applicable imaging approaches for stem cell transplants in patients. We established immediately clinically applicable nanoparticle labeling techniques for tracking stem cell transplants with MR imaging; we have evaluated the long term MR signal effects of iron oxide nanoparticle labeled MASI *in vivo*; and we have defined distinct signal characteristics of labeled viable and apoptotic MASI. This review article will provide an overview over these efforts and discuss important implications for clinical translation.

## Introduction

Arthritis is a major cause of disability, resulting in 992,100 hospitalizations and 44 million outpatient visits in the US each year and causing annual expenses on the order of $95 billion in direct costs (medical treatment) and $47 billion in indirect costs (lost earnings [1,2]). Articular cartilage defects are the key source of pain and functional disability [1-3]. The treatment of cartilage defects provides a major challenge due to the lack of self-regeneration of injured cartilage. New therapies based on transplants of autologous chondrocytes, stem cells, or stem cell derived chondrocytes provide a potentially curative therapeutic option [4]. Advantages of using stem cells for a cell transplant rather than autologous chondrocytes include: (1) one less knee surgery, (2) decreased donor-site morbidity and (3) higher cost effectiveness, while yielding equal or better long term outcomes [5,6]. However, a major barrier for long-term success of cell transplants in cartilage defects is our inability to recognize complications of the engraftment process in a timely manner. To date, a large proportion of transplanted stem cells and chondrocytes undergo apoptosis and/or are cleared from the transplantation site by macrophages [4,7,8]. An imaging method that could visualize and monitor stem cell transplants in cartilage defects directly, non-invasively, and longitudinally *in vivo* would greatly enhance our ability to develop successful cell transplantation techniques. MR imaging is currently the only non-invasive diagnostic test capable of providing high resolution, anatomical and functional information of cartilage defects *in vivo* [9,10]. Over the last 10 years, we have developed non-invasive MR imaging techniques for early detection of complications of the engraftment process of Matrix Associated Stem Cell Transplants (MASI). By exploiting novel, clinically applicable, cell tracking techniques as a new tool to monitor stem cell engraftment outcomes non-invasively *in vivo*, we anticipate significantly improving and accelerating the development of successful therapies for cartilage regeneration in patients, and ultimately, alleviating long term disabilities and related costs to society.

## Cell Labeling with Iron Oxide Nanoparticles

**Figure 1:**
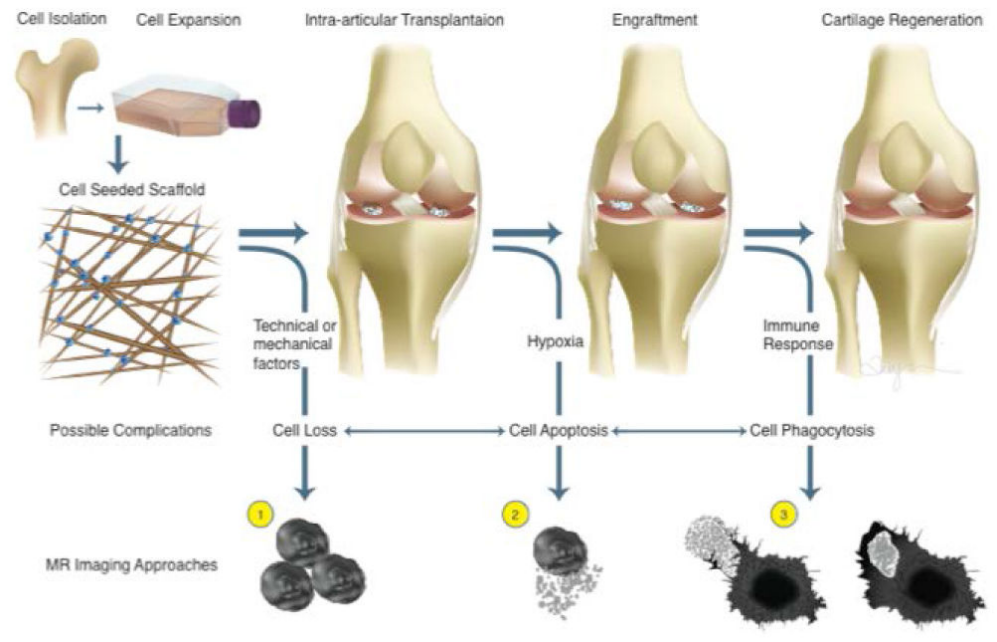
Concept of stem cell-mediated regeneration of osteochondral defects with possible complications and related imaging approaches.

The development of a non-invasive imaging technique for *in vivo* detection of stem cell transplants is crucial for monitoring the safety and efficacy of virtually any stem cell therapy. The ability to non-invasively track transplanted stem cells in vivo, in real time, allows for evaluations of correct stem cell deposition, immediate engraftment patterns, local proliferation, long-term retention at the target site and immune rejection processes (Figure 1). With regards to stem cell transplants in arthritic joints, MR imaging is the only directly clinically applicable imaging modality available for this purpose.

Most cell tracking studies have been performed with iron oxide nanoparticles, because these are easier to introduce into stem cells and provide a higher sensitivity for stem cell detection compared to clinically applicable gadolinium chelates [11-19]. Nanoparticles for MR imaging are categorized based on their size: Superparamagnetic Iron Oxide Nanoparticles (SPIO) with diameters of more than 50 nm are phagocytosedby stem cells in high quantities and therefore, provide highly efficient cell labeling. Conversely, “UltraSmall Superparamagnetic Iron Oxide Nanoparticles” (USPIO) with diameters in the order of 20-50 nm are typically introduced into stem cells via endocytosis and generally provide weaker MR signal effects [20-23,15]. Cell labeling with SPIO is usually possible with simple incubation techniques while efficient cell labeling with USPIO requires transfection techniques [20,21]. Therefore, previous approaches for MR-based cell tracking have been almost exclusively performed with SPIO which allow for easier cell labeling and more sensitive cell detection, such as ferumoxides and ferucarbotran (Feridex™, FDA-approved; Endorem^®^and Resovist™, clinically approved in Europe) [14,15,24-26]. Unfortunately, recently, all clinically applicable SPIO have been taken of the market in the US and in Europe. Major contrast agent companies are developing USPIO as second generation nanoparticles, which offer a wider spectrum of applications and which may have fewer effects on stem cell physiology and differentiation.

**Figure 2:**
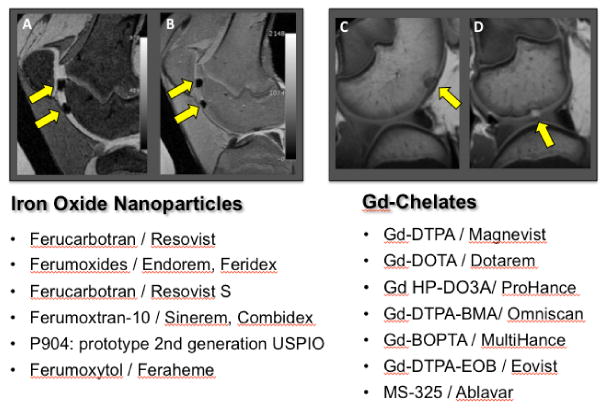
Overview over clinically applicable MR contrast agents which either have been used for clinical cell tracking studies in the past orwhich could, in principle, be applied “off label” for cell tracking studies in patients, because they are FDA approved for other indications. Sagittal T2-weighted (A) and proton-density-weighted (B) MR scans show example of two transplants of iron oxide nanoparticle labeled cells in cartilage defects (left, arrows) and sagittal T1-weighted MR scans (right) show a representative example of an unlabeled (C) or Gd-DTPA labeled (D) transplant (arrows).

A list of clinically applicable MR contrast agents, which have been used or could be used for clinical stem cell tracking applications are listed in Figure 2. Ferumoxytol (Feraheme™) is a USPIO, which has been recently FDA-approved for intravenous treatment of anemia in patients [27-31]. This agent exerts strong signal effects on MR images and can thus be applied “off label” for cell labeling and cell tracking purposes. Ferumoxytol is composed of an iron oxide core and a carboxydextran coat. The agent has a mean hydrodynamic diameter of 30 nm and a high r2 relaxivity of 83 L mmol^-1^ s^-1^ at 20 mHz [32]. We previously applied ferumoxytol as an intravenous contrast agent for MR imaging of arthritis [32] and we performed initial ferumoxytol-labeling experiments of *h*MSCs and other stem cells [23]. Ferumoxytol is currently the only USPIO, which would be immediately available for clinical stem cell tracking applications in the US via an “off label” use. Protamine sulfate can be used as a clinically applicable transfection agent, which can shuttle ferumoxytol into stem cells [33]. Using protamine-transfection, we could detect ferumoxytol-labeled stem cells by a significant negative (dark) signal effect on T2-weighted MR images, which lasted forseveral weeks ([33]; Figure 2). Thu et al. [34] reported a stem cell labeling technique based on ferumoxytol-heparinprotamine complexes, which was more efficient than ours. However, for our application of MASI, our clinicians requested a “heparin-free” labeling technique, in order to avoid potential secondary bleeds or heparin-induced cartilage damage [35]. With our protocol, this potential side effect of heparin, delivered with labeled cells, would be excluded.

**Figure 3:**
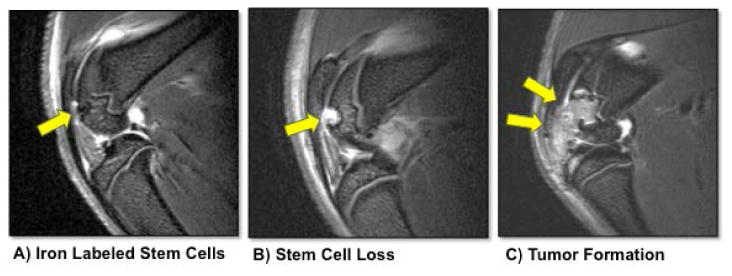
Sagittal T2-weighted MR images of rat knee joints with an osteochondral defect of the distal femur (arrow) and status post implantation of a ferumoxitol-labeled stem cell transplant (A), loss of labeled cells from the transplant site (B) and tumor formation (C).

Both SPIO-labeling and ferumoxytol-labeling results in lysosomal retention of iron oxide nanoparticles in the cells' cytoplasm, where they undergo slow iron metabolism over several weeks [20,24,36]. The labeled cells provide a strong, significant, negative (dark) signal effect on MR images, *in vitro* and *in vivo* [20,15,24,36,37]. We have shown that optimized protocols for nanoparticle labeling do not impair the viability or differentiation capacity of iron oxide labeled stem cells: While exposing stem cells to excessive amounts of iron oxides has impaired stem cell differentiation, particularly chondrogenesis, labeling stem cells with limited quantities of iron oxide nanoparticles had no apparent effect on stem cell viability, proliferation or differentiation [24,38]. Our team developed stem cell labeling protocols that provide a compromise between cellular iron load that allows MR detection (the higher the better) and cellular iron load that ensures preserved stem cell function and differentiation capacity (the lower the better). Optimized cell labeling protocols lead to an unimpaired chondrogenesis of *h*MSCs when compared to unlabeled controls [15,24]. In general, an iron load of less than 10 picogram per cell has not shown any impairment in chondrogenesis in our experience, although this would have to be confirmed for other cell types. Ex vivo cell labeling could be used to diagnose correct stem cell deposition or stem cell loss from the target site, and it could also help detecting *in vivo* tumor formation based on observations of expanding cell deposits and too fast dilution of the iron label (Figure 3). Depending on transplanted cell type, viable cell transplants metabolize the iron label within 2-4 weeks. Therefore, the iron oxide induced MR signal loss of stem cell transplants at 0-4 weeks after MASI does usually not interfere with more long term indicators of cartilage defect repair, defined by the MOCART score (magnetic resonance observation of cartilage repair tissue: “defect fill,” “cartilage interface,” “surface,” “adhesions,” “structure,” “signal intensity,” “subchondral lamina,” and “effusion”, [39,40]). Future studies have to show, if abnormal MR signal kinetics of iron labeled stem cells, such as early loss of iron signal at the transplant site, correlate with late findings of incomplete or failed cartilage repair, as defined by the MOCART score.

## *In Vivo* Labeling of Bone Marrow Derived Stem Cells

As of today, seven clinical trials have been reported, in which researchers tracked *ex vivo* labeled cells in patients [41]. However, due to stringent FDA-controlled regulatory issues, all of these studies have been performed outside the United States [41]. Furthermore, first generation iron oxide nanoparticles (Feridex orEndorem), which had been used for *ex vivo* stem cell labeling for these previously reported trials, have been discontinued by the pharmaceutical industry due to economical reasons. The limited biodistribution of SPIO lead to limited applications and limited revenues. As outlined above, our group and others used the ultrasmall SPIO (USPIO) ferumoxytol (Feraheme) as an alternative stem cell label. However, the combination of “off label” use of several drugs needed for *ex vivo* cell labeling together with cell manipulations (such as cell washing to remove excess label) represents a hurdle for FDA-approval and clinical translation in the United States. We solved this problem by labeling mesenchymal stem cells (MSC) *in vivo*, via a simple intravenous injection of ferumoxytol, which is endocytosed by MSC in bone marrow [42]. After their harvest from bone marrow and transplantation into arthritic joints, the iron labeled MSC could be tracked with clinical MR imaging tools. This new procedure does not require any alteration of stem cell harvest or transplantation procedures, does not involve any *ex vivo* stem cell manipulation, is immediately clinically translatable via “off label” use of the FDA-approved iron supplement ferumoxytol and could be widely utilized for numerous novel stem cell therapies currently entering clinical trials. The ability to directly track ferumoxytol labeled MSC *in vivo* could enable a wide variety of tissue regeneration approaches and stem cell imaging applications beyond arthritis research.

## Differentiating Viable and Dead Stem Cell Transplants

Another complication of a stem-cell transplant is stem cell loss due to cell death. This often arises when cells are moved from a nurturing cell culture environment into hostile *in vivo* environments of osteochondral injuries. While the *in vitro* environment provides stable temperature, oxygen levels, pH, and nutrition conditions, an osteochondral defect is characterised by a hypoperfused, hypoxic environment [43,44], with high levels of inflammatory mediators [45]. As a result, a large portion of implanted cells undergo apoptosis [46] and are cleared from the transplantation site by macrophages, [4,7,8], which leads to incomplete repair [47]. If we were able to determine cell viability and cell death with an imaging test, then we could better develop MASI approaches that lead to successful cartilage regeneration outcomes.

Our studies have shown that viable and apoptotic MASI demonstrate distinct signal characteristics on MR images, when the cells are labeled with iron oxide nanoparticles [15,36,37,48]. Iron oxide labeled viable MASI demonstrated an increasing area of T2-signal loss in the early post-transplant period (1-2 weeks post MASI), which correlated to stem cell expansion over a larger areaat the transplantation site. The cells persisted in the defect for about 1-2 weeks, as proven by MRI and histopathology [15]. By contrast, transplants of iron oxides labeled apoptotic stem cells did not expand and demonstrated a central loss of T2-signal on follow up MR imaging studies due to loss of stem cells from the transplantation site and lack of repair of the defect [15]. Histopathology confirmed persistence of viable labelled and unlabeled *h*MSCs in the defects for up to 2 weeks and repair of the underlying defect, while transplantation of apoptotic *h*MSCs lead to faster cell elimination from the transplant site via macrophage phagocytosis and resulted inpersistent cartilage defects [15].

## Imaging Technique to Track Interactions of Host Macrophages with MASI

Death of transplanted stem cells could be also detected indirectly, based on host immune responses [4,49]. Proteins released from apoptotic stem cells serve as a chemotactic factors for bone marrow macrophages, which migrate to and home in MASI, where they phagocytose the dead cells [15]. Converseley, Arinzeh et al. [29] showed that successful bone regeneration was not associated with significant immune cell migration into the transplant. An imaging technique that could visualize macrophage migrations into MASI could help to detect stem cell death and monitor the effect of supportive factors, such as scaffolds, growth factors and immune modifiers on stem cell engraftment outcomes. We have shown in preclinical and clinical investigations, that bone marrow macrophages can be labeled *in vivo* with USPIO and that migration of ferumoxytol-labeled macrophages into MASI can be detected with MR imaging [50-52]. The approach relies on pre-loading bone marrow macrophages via intravenous ferumoxytol administration, followed by transplantation of unlabeled stem cells. Iron oxide labeling of bone marrow macrophages allows for detection of macrophage migration into unlabeled MASI. Our data have shown that bone marrow macrophages migrate in larger quantities into apoptotic MASI as opposed to viable MASI [42]. Since this approach relies on “off label” use of an FDA-approved drug, without changing route or dose of administration, this new procedure could likely be more easily translated to the clinic than *ex vivo* labeling procedures described above, and thereby directly benefit stem cell therapies currently entering clinical trials.

## Safety and Practical Considerations for Clinical Translation

We have applied various iron oxide nanoparticle compounds as MR contrast agents in clinical trials [50,52-58]. These agents are generally well tolerated and show excellent safety profiles [28,31,52,56,57]. The delivered iron dose for potential ferumoxytol cell tracking studies in patients would be less than 1 mg Fe (Note: these are coated iron oxide nanoparticles, not free iron), which is considerably less than the currently administered dose for anemia treatment (5 mg/kg) or the iron dose administered with one blood transfusion. Iron oxide nanoparticles, which have been released from apoptotic cells, are either phagocytosed and metabolized by resident macrophages or they are absorbed by the synovium, enter the blood and are slowly metabolized by macrophages in liver and spleen [15,26,32,59]. Ferumoxytol is not excreted via the kidneys and not associated with any risk of nephrogenic sclerosis (a potential adverse event with Gd-chelates) [30,31,60]. In fact, ferumoxytol has been FDA-approved for the treatment of anemia in patients with renal insufficiency and has been safely applied in more than 150,000 patients to date. Anaphylaxis or anaphylactoid reactions with ferumoxytol were reported in 0.1-0.2% of exposed subjects, which is comparable to other MR contrast agents [30,31,60]. Our group uses ferumoxytol « off label » via an IND with the FDA as an intravenous contrast agent for patients with bone tumors. Results from these clinical trials will provide valuable information about contrast agent signal characteristics and safety in patients. We plan to use this experience towards clinical translation of our cell tracking techniques.

Most studies on stem cell-mediated cartilage repair have been performed with either chondrocytes or MSC. Alternative cell types comprise Adipose Derived Fat Cells (ADSC), synovial and periosteal derived stem cells. The ideal cell source for MASI should allow easy and inexpensive cell harvesting, expansion and maintenance, involve minimal donor morbidity, contribute directly to chondrogenic matrix regeneration and have a low risk of immune responses or transmission of other diseases. MSC represent very heterogenous cell populations and it is highly debated, wether these cells contribute directly to chondrogenic matrix regeneration orif they rather cause a supportive microenvironment for cartilage defect repair [61]. While using autologous stem cells for cartilage repair would be desirable to minimize host immune responses, the yield of MSC which could be retrieved from bone marrow aspirates and which are capable of forming cartilage would likely be too low for most « one-stop-shop » approaches and require *ex vivo* enrichment and expansion of cartilage forming phenotypes (positive for CD105, CD73, CD90 and negative for CD45, CD34, CD11b, CD19, HLA-DR; according to the International Society for Cell Therapy; ISCT). Using conventional separation technologies, 5 ml of human unprocessed bone marrow produces approximately 250 million mononuclear cells ±90 million. One in 100,000=2500 of these cells are cartilage-forming MSC as defined above. In an outpatient procedure, up to about 180 mlof bone marrow aspirate or 90,000 MSC could be retrieved. In an inpatient procedure, 10-15 ml/kg could be harvested, which would translate into 10 cc/kg=350,000 MSC for a 70 kg patient. These numbers are too low for most cartilage regeneration needs. Since cell culture expansion could lead to changes in cell phenotypes and cell de-differentiation, « off the shelf » cell products are preferred by many investigators because of their immediate and potentially unlimited availability and better characterization. However, these products will require close observations of potential host immune responses that could lead to rejection of the transplant.

Another barrier for clinical translation is the limited availability of immediately clinically applicable scaffolds. Hydrogel-based bioengineered scaffolds have shown very promising results in preclinical studies. However these products are not FDA-approved and thus, not clinically available within the foreseeable future [61]. Table 1 provides a list of FDA-approved scaffolds which would be potentially more immediately clinically applicable via an « off label » use. Scaffold features that affect stem cell engraftment outcomes and which have to be evaluated for every new scaffold product include the following [62-64]: (1) Biocompatibility; Ability of the scaffold material to integrate into living tissue. (2) Mechanical property: Resistance to local mechanical forces. (3) Pore size: Influences cell aggregation and differentiation. (4) Structure and geometry: Influences proliferation and differentiation of the transplanted cells. (5) Biodegradation property: Fast or slow elimination from the body. (6) Biochemical integration: Long term integration and availability of growth factors or cytokines. Clinic-to-bench-and-back research is urgently needed to evaluate the effect of various stem-cell scaffold compositions on bone and cartilage regeneration outcomes.

## Summary

Clinically applicable cell tracking techniques will enable us to overcome the bottle neck of diagnosing stem cell transplant failures, avoid long term and invasive follow up studies of lost or dead transplants and help to assign patients with transplant failure to early interventions or alternative treatment options. Clinical translation of the described imaging techniques could help to direct patients with apoptotic and/or lost MASI, as diagnosed by cellular MR imaging, to repeated or alternative treatments. On the other hand, patients with successful transplants could be spared from invasive follow up studies. Cellular imaging tools could be also utilized to study the effect of new types of cell transplants (e.g. chondrocytes or chondrogenic precursors derived from induced pluripotent stem cells or embryonic stem cells [65]), scaffolds, growth factors and immune response modifiers on MASI engraftment outcomes, which could in turn inform the development of more successful MASI approaches. Since clinical trials of new combination therapies are expensive and take years to complete, the impact of our imaging technique could be immense. Our cellular imaging techniques would be in principle readily clinically applicable, could help to improve and tailor individualized therapeutic approaches, and ultimately, improve successful joint regeneration and long term outcomes.

## Figures and Tables

**Table 1:** Overview over clinically applicable scaffolds.

Product Name	Company	Primary Indication	Composition
**INTEGRA™ FlowableWound Matrix**	Integra	Wound healing	Granulated cross-linked bovme tendon collagen and glycosaminoglycan
**lnf orce^®^Reinforcement Matrix**	Integra	Tendon repair	Cross-linked porcine derived collagen I
**SurgiMend**	TEl Biosciences	Soft tissue repair	Acellular dermal Collagen matrix derived from fetal and neonatal bovine dermis
**AlloPatch HD™**	MTF Sports Medicine	Tendon augmentation	Acellular Human Dermis
**FlexHo®**	Ethicon	Hernia repair breast reconstruction	Acellular dermal matrix derived from donated human allograft skin
**BioFiber'™**	Tornier	Soft tissue repair	Absorbable biologic polymer
**TRUFITTM CB Plug**	Smith & Nephew	Cartilage repair	Polymers, ceramics and fibers
**ChondroMimetic™**	TiGenix	Cartilage repair	Readily absorbed, non-synthetic, collagen, GAG, calcium phosphate
**OseoFit**	Kensev Nash	Cartilage repair	Collagen formulation1 synthetic polymer and ceramic
**Matrix Collagen Particles**	Collagen Matrix	Wound healing	Type I collagen
**Collagen Sponge**	Collagen Matrix	Wound healing	Type I collagen
**Collagen Film**	Collagen Matrix	Wound healing	Type I collagen
**Collagen Film/Collieva**	lnnocoll	Wound healing	Purified type 1 collagen
**Pelvicol^®^Acellular Collagen Matrix**	C.R. Bard	Soft tissue repair	Cross-linked Porcine dermal collagen
**ARTISS**	Baxter Healthcare Corporation	Wound healing	.Human fibrinogen and a synthetic fibrinolysis inhibitor / low-concentration human thrombin solution In a calcium chloride solution
**TISSEEL**	Baxter Healthcare	Hemostasis / Sealine	Human fibrinogen and a synthetic fibrinolysis inhibitor, aprotinin
**EVICEL^®^Fibrin Sealont**	Ethicon360	Hemostasis	The only all human, aprotinin free, fibrin sealant
